# Milk Sickness

**Published:** 1841-07

**Authors:** 


### MILK SICKNESS.

The "*Mill Stone Knob*" in Sumner county, Tenn., is one of the localities which have been famed, since the settlement of the country, for giving rise to milk-sickness. It is now generally called the "*poison* knob." Yet domestic animals of every description may be seen, at this season, feeding upon the grass which grows luxuriantly on its sides wherever the sun has gained admission to the soil, and it is understood that they may continue to range it with safety until about the commencement of winter. In a hasty excursion which we made to the knob, a few weeks since, we saw, every now and then, the spot where a fire had been kindled to consume the carcass of some animal which had perished of the poison. Such occurrences, we learnt, were still not uncommon in the neighborhood, yet owing to the caution of the people in keeping their milch-cows upon pastures which have been cultivated, a case of poisoning in the human subject has not occurred for three years. The country around the knob is thickly settled, and families are found living on the sides of the hill. They feel safe so long as they abstain from the flesh and milk of animals that have been ranging the "poison knob," or in other words, while they confine their stock to their own cultivated grounds. This knob is one of the most fertile spots to be seen in that fertile region of country, and has a black, rich soil to its very summit, which is now covered with a luxuriant growth of cane. So fine is the pasture upon it, in winter, that persons drive their young cattle to it to pass that season. A few die of the poison, but they feel themselves indemnified in the saving of forage and labor that would be necessary to take the survivors through the winter. I need hardly add, that the cause of this singular disease has not been discovered. The *remedy* for it is *prevention*, and this consists in bringing the soil under the dominion of the plough. One year's cultivation effectually eradicates the poison, and forever afterwards the lands infected by it, if sowed in grass, may be depastured with impunity. It is a fact attested by all with whom I conversed, that hogs and buzzards, as well as hens and turkeys, are poisoned by the flesh of animals that have died from this cause. Dr. Graffe, it may be known to some of the readers of this Journal, came to a contrary conclusion after some experiments upon the hog, as related in a late number of the Medical Examiner. Y.

